# Development and validation of a novel nomogram to predict overall survival of patients with moderate to severe chronic kidney disease

**DOI:** 10.1080/0886022X.2022.2032744

**Published:** 2022-02-15

**Authors:** Ning Li, Guowei Zhou, Yawei Zheng, Enchao Zhou, Weiming He, Wei Sun, Lu Zhang

**Affiliations:** Affiliated Hospital of Nanjing University of Chinese Medicine, Jiangsu Province Hospital of Chinese Medicine, Nanjing, PR China

**Keywords:** Chronic kidney disease, overall survival, nomogram, mortality, clinical application

## Abstract

**Introduction:**

The risk of death significantly increased from stage 3 chronic kidney disease (CKD) onward. We aimed to construct a novel nomogram to predict the overall survival (OS) of patients afflicted with CKD from stage 3–5.

**Methods:**

A total of 882 patients with stage 3–5 CKD were enrolled from the NHANES 2001–2004 survey. Data sets from the 2003–2004 survey population were used to develop a nomogram that would predict the risk of OS. The 2001–2002 survey population was used to validate the nomogram. Least absolute shrinkage and selection operator (Lasso) regression was conducted to screen the significant predictors relative to all-cause death. The multivariate Cox regression based on the screened factors was applied to effectively construct the nomogram. The performance of the nomogram was evaluated according to the C-index, the area under the receiver operating characteristic curve (AUC), and the calibration curve with 1000 bootstraps resample. Kaplan–Meier’s curves were used for testing the discrimination of the prediction model.

**Results:**

Five variables (age, urinary albumin-to-creatinine ratio (UACR), potassium, cystatin C (Cys C), and homocysteine) were screened by the Lasso regression. The nomogram was constructed using these factors, as well as the CKD stage. The included factors (age, CKD stage, UACR, potassium, Cys C, and homocysteine) were all significantly related to the death of CKD patients, according to the multivariate Cox regression analysis. The internal validation showed that this nomogram demonstrates good discrimination and calibration (adjusted C-index: 0.70; AUC of 3-, 5-, and 10-year OS were 0.75, 0.78, and 0.77, respectively). External validation also demonstrated exceedingly similar results (C-index: 0.72, 95% CI: 0.69–0.76; AUC of 3-, 5-, and 10-year OS were 0.76, 0.79, and 0.80, respectively).

**Conclusions:**

This study effectively constructed a novel nomogram that incorporates CKD stage, age, UACR, potassium, Cys C, and homocysteine, which can be conveniently used to facilitate the individualized prediction of survival probability in patients with stage 3–5 CKD. It displays valuable potential for clinical application.

## 1. Introduction

Chronic kidney disease (CKD) has become a major global health concern [1]. Currently, nearly 700 million people worldwide have suffered from CKD. The developing burden placed on public health facilities is jointly observed with that of the profound economic burden experienced in various communities across the globe. It is well established that CKD progression is associated with undesirable clinical outcomes, such as cardiovascular complications, infections, and malignancies [2–4]. Currently, CKD is acknowledged as the 18th leading cause of death globally [5], displaying an 82% increase in the absolute number of deaths in the past two decades [6]. The current consensus is that the mortality risk of patients with CKD has significantly increased since stage 3 [7–9]. However, in consideration of the high heterogeneity of these patients, it is difficult to accurately predict the prognosis of individual survival. Given the challenges pertaining to the current situation, the development of an effective nomogram would be of benefit in addressing this concern.

Nowadays, nomograms have been widely applied to assess the prognosis of complex diseases [10,11]. Nomograms typically incorporate the laboratory parameters and clinical signs of patients, integrating the contributions of various predictors in order to realistically forecast the probability of certain endpoint outcomes. Through this fundamental action, nomograms play a significant role in the drive toward personalized medicine. Regrettably, no nomogram has been constructed to predict the survival outcome in patients with moderate to severe CKD. The construction of an accurate prediction model is helpful for nephrologists to screen and identify high-risk patients so that they may provide timely intervention to improve prognosis. Therefore, this study aims to construct a visualized prediction model, scoring the probability of 3-years overall survival (OS), 5-years OS and 10-years OS via nomogram in CKD patients.

## 2. Methods

### 2.1. Patients and predictors

The data of enrolled patients were based on the 2001–2004 National Health and Nutrition Examination Survey (NHANES). We selected those four years because the information of patients was comparatively completed. Prior to data collection, the study procedures of NHANES had been approved by the Institutional Review Board (IRB) of the National Center for Health Statistics (NCHS), with written informed consent obtained. We selected the population in 2003–2004 as the training cohort, and the population in 2001–2002 as the external validation cohort. Patients were enrolled if they were over 18 years old with stage 3–5 CKD (defined as estimated glomerular filtration rate (eGFR)<60 mL/min per 1.73 m^2^). The calculation of eGFR was based on the formula of CKD-EPI, and measured using the average of the two repeat measurements. Patients with incomplete clinical data or lost contact in the follow-up period were excluded. The predictors we extracted included cystatin C (Cys C), gender, age, glycosylated hemoglobin (HbA1c), diabetes, blood urea nitrogen (BUN), bicarbonate, serum phosphorus, serum potassium, serum sodium, serum uric acid (UA), hypertension, urinary albumin-to-creatinine ratio (UACR), C-reactive protein (CRP), homocysteine, eGFR, and anemia (defined as hemoglobin <13 g/dL for men and <12 g/dL for women). The stage division of CKD and UACR is based on the recommendation of KDIGO guidelines [12] (CKD stage – stage 3a: eGFR 45–59 mL/min per 1.73 m^2^; stage 3b: eGFR 30–44 mL/min per 1.73 m^2^; stage 4: eGFR 15–29 mL/min per 1.73 m^2^; stage 5: eGFR 0–14 mL/min per 1.73 m^2^. UACR: normal: <30 mg/g; microalbuminuria: 30–299 mg/g; macroalbuminuria: ≥300 mg/g). The presence of complications was obtained through the questionnaire.

### 2.2. Outcome

The endpoint outcome of this study was defined as all-causes of death. In the nomogram, we used the probability of OS in 3-years, 5-years, and 10 years, respectively instead. The status of death and the time of follow-up were extracted through the public-use linked mortality file obtained from the NCHS and matched with the ID of participants from the NHANES database. The time of OS was determined as the interval between the date of the interview and the date of death or the last follow-up date.

### 2.3. Statistical analysis

The Shapiro–Wilk method was used to test the continuous data. The continuous variables that conformed to normal distribution were compared by independent samples *t*-test and displayed as mean ± standard deviation (SD). Variables that were not normally distributed were tested by the Mann–Whitney’s *U* test and reported as median, (1st–3rd quartile). Chi-square tests were used to compare categorical variables. If the theoretical frequency was less than 10, Fisher's exact test was preferred. For continuous variables, we grouped these with clinical experience or the cutoff value (Kaplan–Meier’s method). The missing covariate and dependent variable data were excluded from the analysis. To avoid the collinearity of inclusion covariates, the independent prognostic factors were screened by the least absolute shrinkage and selection operator (Lasso) regression [13]. The predictors selected by the Lasso regression were incorporated into a Cox regression to build the nomogram. The receiver operating characteristic (ROC) curves were used to test the sensitivity and specialty of the model [14]. The predictive performance of the nomogram was measured by a concordance index (C index) and calibration curve. The 1000 bootstrap resamples validation method was used for internal validation [15]. To further determine the discrimination of the nomogram, we calculated the total scores of each patient, and then stratified them into two groups based on the median scores, and performed survival analysis via the Kaplan–Meier method. Decision curve analysis (DCA) was conducted to determine the clinical usefulness of the nomogram by quantifying the net benefits at different threshold probabilities [16]. For external validation, we calculated the total point of each patient according to an established nomogram and used it as a factor to perform the Cox regression. Finally, the C-index, as well as the calibration curve of the validation cohort, was derived. In all analyses, *p*<.05 was considered statistically significant.

All statistical analyses were performed using EmpowerStats and R software version 4.05 (https://www.rproject.org/). Study population description and Lasso regression were implemented through EmpowerStats. The group of continuous variables (survminer package), construction of prediction model (rms Package), nomogram, ROC curve (timeROC), C-index (rms package), and calibration plot were implemented through R software.

## 3. Results

### 3.1. Baseline characteristics and follow-up

According to our prespecified inclusion and exclusion criteria, a total of 882 patients with moderate to severe renal impairment were enrolled in this study. Overall, 508 patients died. The median survival times were 122 months (range, 1–152), and median follow-up times were 143 months (range, 133–157). The mortality rate was 13.18% in 3 years, 22.02% in 5-years, and 48.9% in 10-years. Among the enrolled population in the training cohort, 55.42% were male, 74.01% of cases were complicated with diabetes, and 67.15% of cases were complicated with hypertension. The median eGFR was 51.05 mL/min per 1.73 m^2^, and the median UACR was 10.48 mg/g. In the validation cohort, 53.05% were male, 75.91% complicated with diabetes, and 64.94% complicated with hypertension. The median eGFR was 52.50 mL/min per 1.73 m^2^, and the median UACR was 10.00 mg/g. The laboratory and clinical characteristics of the patients are shown in Table 1.

**Table 1.** The clinicopathologic characteristics of patients in the training and validation cohorts.

Parameters	Training cohort (*n* = 554)	Validation cohort (*n* = 328)	*p* Value
Cystatin-C (mg/L)	1.23 ± 0.51	1.20 ± 0.55	.439
Age (years)	72.47 ± 12.26	72.59 ± 11.92	.887
HbA1c (%)	5.60 (5.40–6.00)	6.00 (5.00–6.00)	.043
Hemoglobin (g/dL)	13.71 ± 1.52	13.88 ± 1.53	.124
Blood urea nitrogen (mmol/L)	6.78 (5.00–8.57)	7.00 (5.00–9.00)	.972
Bicarbonate (mmol/L)	25.00 (24.00–27.00)	25.00 (24.00–27.00)	.839
Phosphorus (mmol/L)	1.22 ± 0.18	1.05 ± 0.23	<.001
Uric acid (µmol/L)	374.70 (315.20–434.20)	369.00 (309.00–440.00)	.977
Potassium (mmol/L)	4.15 ± 0.43	4.19 ± 0.51	.288
UACR (mg/g)	10.48 (5.62–33.00)	10.00 (5.00–29.00)	.514
C-reactive protein (mg/dL)	0.26 (0.13–0.60)	0.00 (0.00–1.00)	<.001
Homocysteine (umol/L)	13.66 ± 5.12	13.80 ± 5.56	.692
eGFR (mL/min per 1.73 m^2^)	51.05 (43.62–55.91)	52.50 (44.00–56.00)	.274
Gender (*n*/%)			.495
Women	247 (44.58%)	154 (46.95%)	
Man	307 (55.42%)	174 (53.05%)	
Diabetes (*n*/%)			.529
No	410 (74.01%)	249 (75.91%)	
Yes	144 (25.99%)	79 (24.09%)	
Hypertension (*n*/%)			.502
No	182 (32.85%)	115 (35.06%)	
Yes	372 (67.15%)	213 (64.94%)	
Anemia (*n*/%)			.48
No	463 (83.57%)	280 (85.37%)	
Yes	91 (16.43%)	48 (14.63%)	
Death (*n*/%)			.769
No	237 (42.78%)	137 (41.77%)	
Yes	317 (57.22%)	191 (58.23%)	

UACR: urinary albumin-to-creatinine ratio; eGFR: estimated glomerular filtration rate.

### 3.2. Lasso Cox and multivariate analysis of the clinical indicators

**Figure 1.**
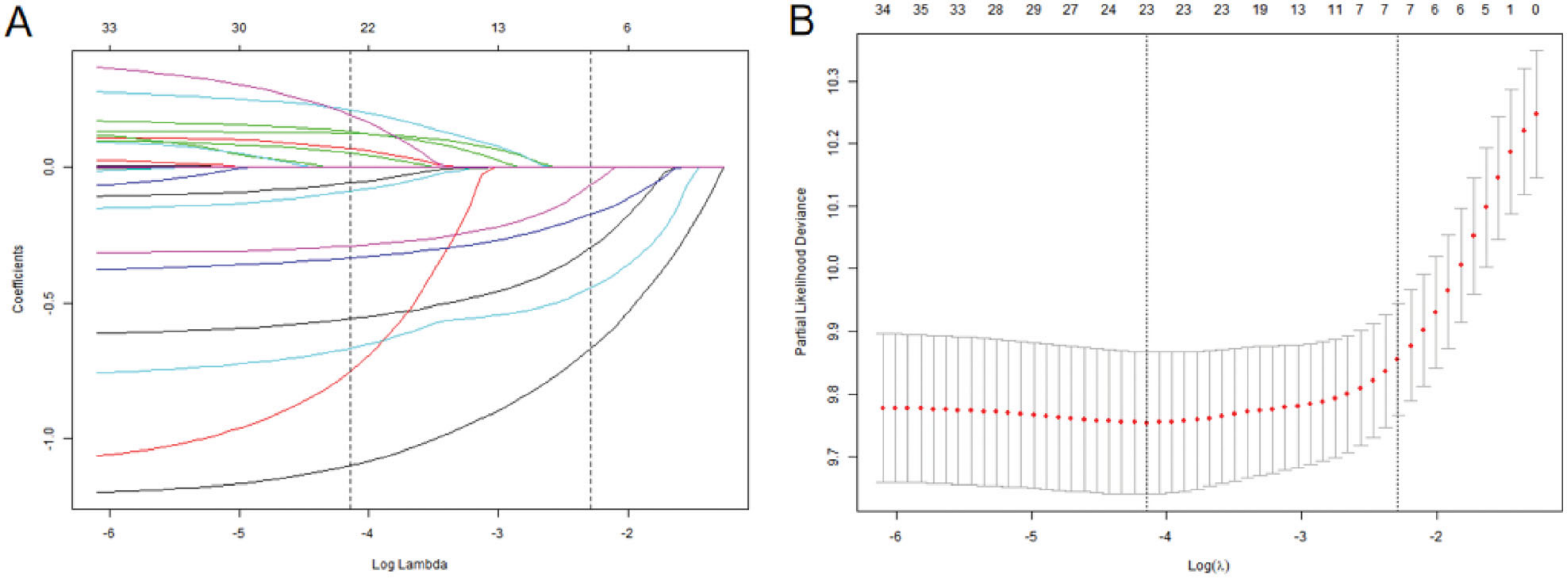
The predictor selecting process by the Lasso Cox regression model. (A) Lasso coefficients of a total 17 clinical indicators. (B) .log (lambda) and partial likelihood deviance were shown; the dotted line is displayed at the minimum log (lambda) and represents the optimal number of predictors.

A total of 17 variables were included in the Lasso Cox analysis (Figure 1) to avoid collinearity and confounding factors. Five variables (UACR, Cys C, age, potassium, and homocysteine) were left with nonzero coefficients according to the minimum criteria. Multivariate Cox regression was conducted to construct the survival prediction model. The results show that all variables (Cys C: HR: 1.75, 95% CI: 1.25–2.45; age: HR: 3.37, 95% CI: 2.34–4.86; potassium: 1.46, 95% CI: 1.14–1.87; homocysteine: HR: 1.44, 95% CI: 1.11–1.87; UACR (reference normal): microalbuminuria HR: 1.78, 95% CI: 1.36–2.33, macroalbuminuria HR: 1.91, 95% CI: 1.27–2.85; CKD stage (reference stage 3a): stage 3b HR: 1.03, 95% CI: 0.77–1.38, stage 4 HR: 1.15, 95% CI: 0.76–1.73, stage 5 HR: 3.74, 95% CI: 1.27–10.98) were significantly co-related with all-causes of death (Table 2).

**Table 2.** Multivariate Cox regression analyses of variables.

Variables	*β*	Hazard ratio	95% CI (lower)	95% CI (upper)	*p* Value
Cystatin-C (mg/L)					
≤1.1	ref	ref	–	–	–
>1.1	0.56	1.75	1.25	2.45	.001
Age (years)					
≤65	ref	ref	–	–	–
>65	1.22	3.37	2.34	4.86	<.001
Potassium (mmol/L)					
≤5	ref	ref	–	–	–
>5	0.38	1.46	1.14	1.87	.003
Homocysteine (umol/L)					
≤12	ref	ref	–	–	–
>12	0.36	1.44	1.11	1.87	.006
CKD stage					
Stage 3a	ref	ref	–	–	–
Stage 3b	0.03	1.03	0.77	1.38	.859
Stage 4	0.14	1.15	0.76	1.73	.512
Stage 5	1.32	3.74	1.27	10.98	.017
UACR (mg/g)					
<30	ref	ref	–	–	–
30–299	0.58	1.78	1.36	2.33	<.001
≥300	0.66	1.91	1.27	2.85	.002

UACR: urinary albumin-to-creatinine ratio; CKD: chronic kidney disease.

### 3.3. Development and assessment of predictive nomogram

#### 3.3.1. Internal validation

**Figure 2.**
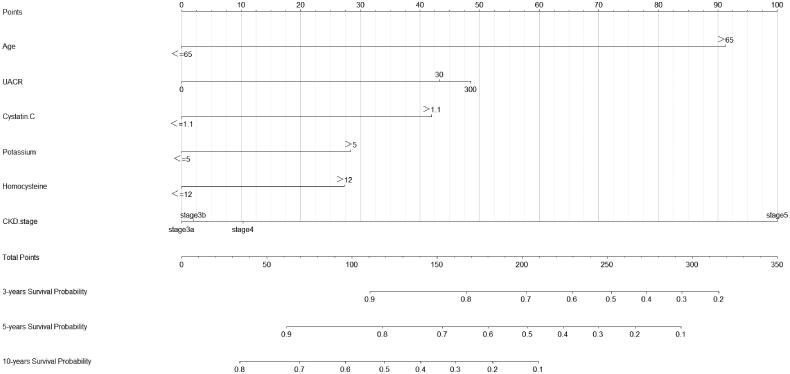
Nomogram to predict the probability of overall survival in patients with moderate to severe CKD. UACR: urinary albumin-to-creatinine ratio; CKD: chronic kidney disease.

**Figure 3.**
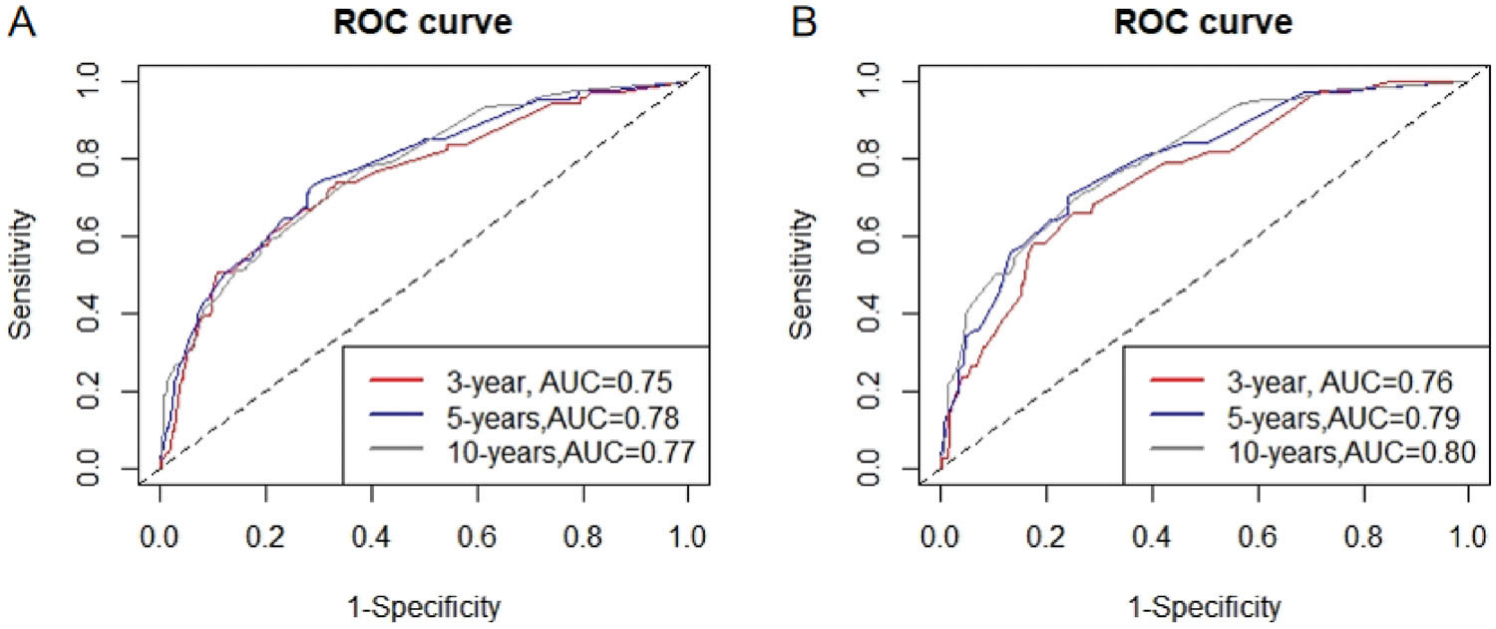
Nomogram ROC curves. (A) ROC curve of the nomogram in the training cohort; (B) ROC curve of the nomogram in the validation cohort.

**Figure 4.**
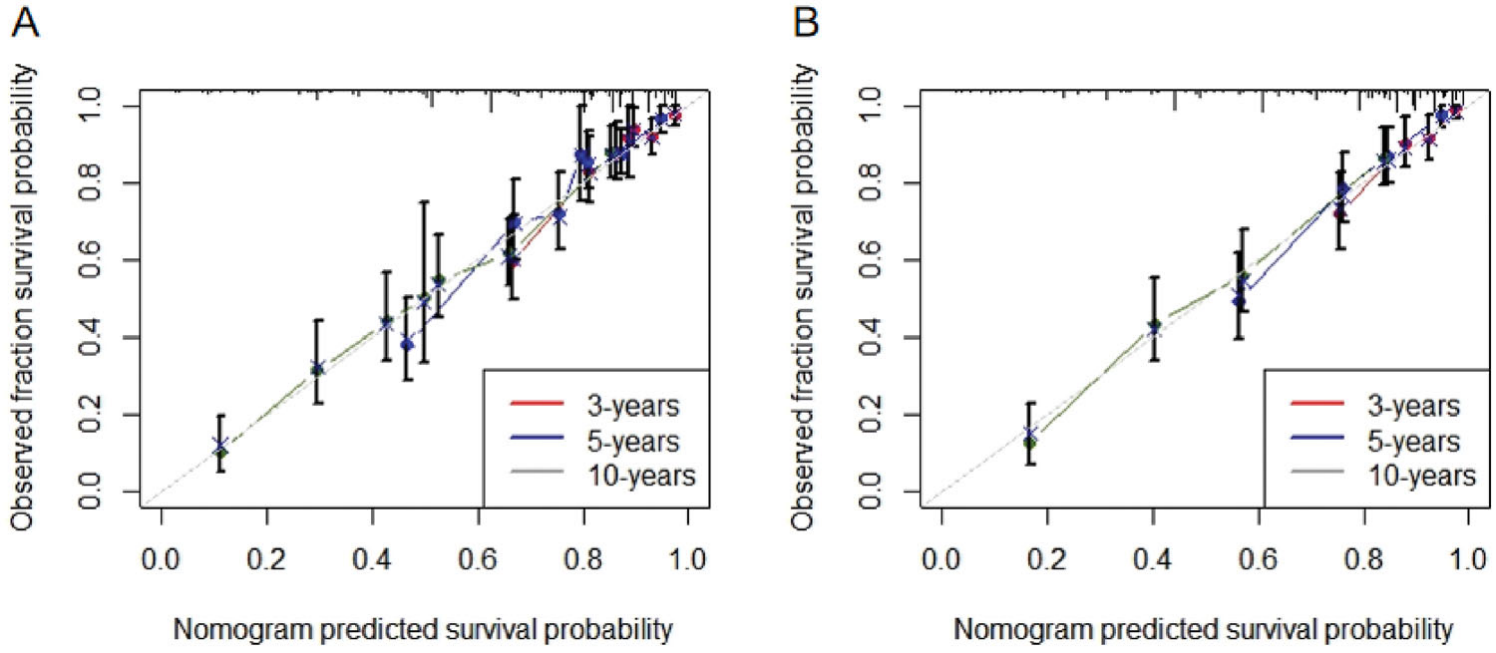
Nomogram calibration curves. (A) Calibration curve of the nomogram in the training cohort; (B) calibration curve of the nomogram in the validation cohort.

**Figure 5.**
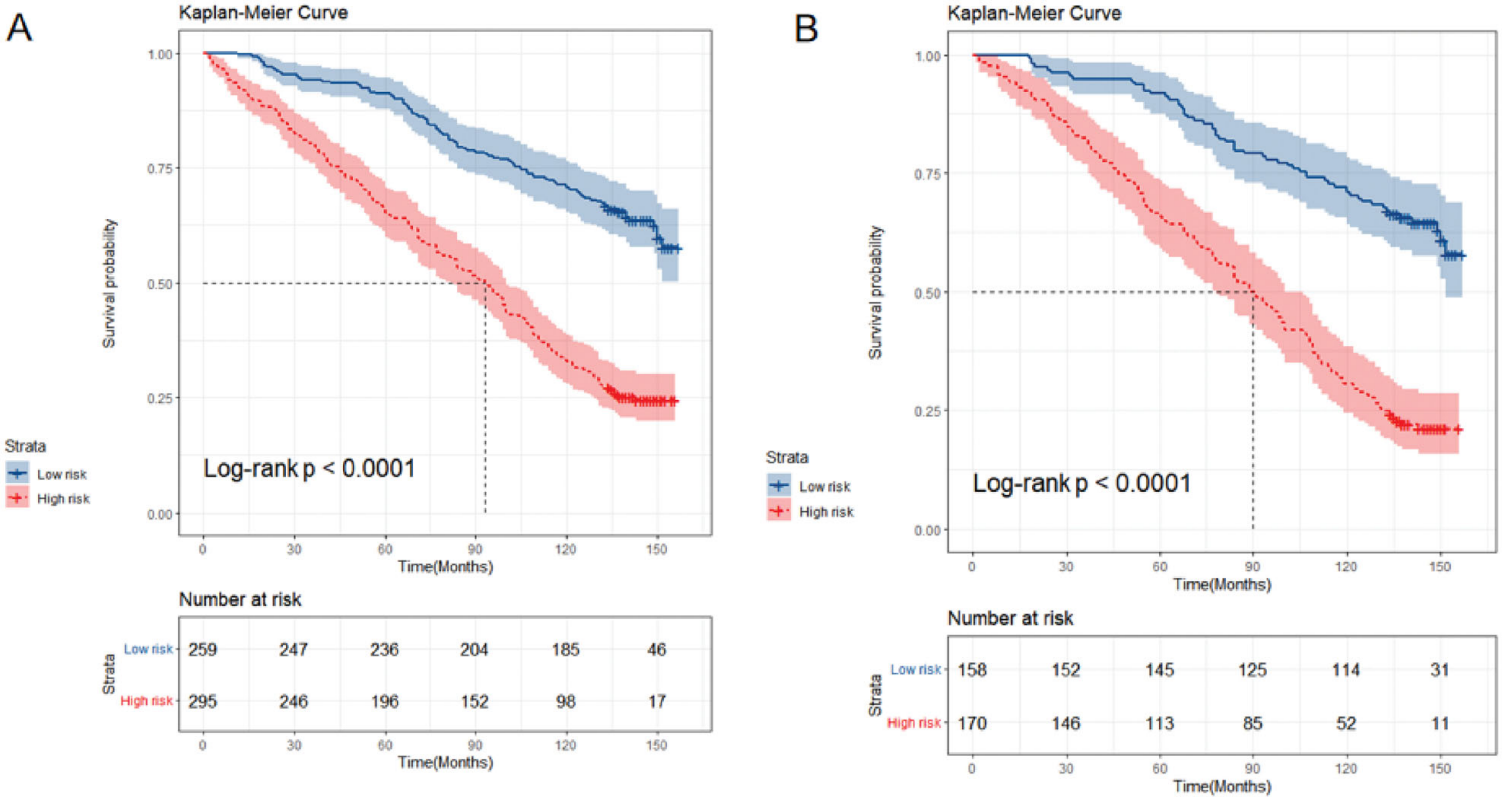
Kaplan–Meier’s curve of overall survival in the training cohort and validation cohort. (A) Training cohort; (B) validation cohort.

**Figure 6.**
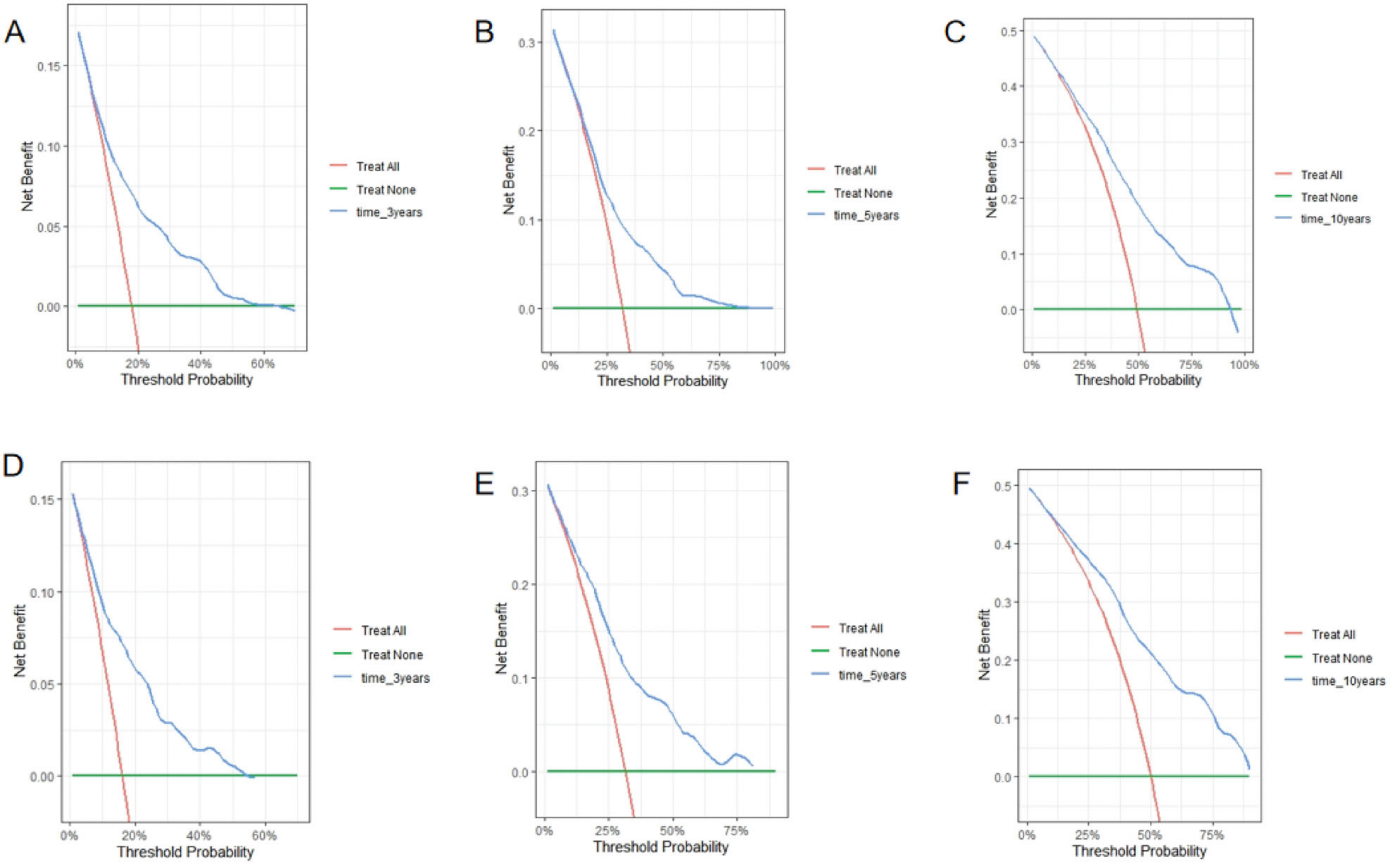
Nomogram decision curve analyses. Decision curve of the nomogram to predict 3-year (A), 5-year (C), and 10-year (E) in the training cohort, and 3-year (B), 5-year (D), and 10-year (F) in the validation cohort.

We developed a predictive nomogram (Figure 2) containing age, CKD stage, UACR, Cys C, potassium, and homocysteine. The area under the receiver operating characteristic curve (AUC) of 3-years, 5-years, and 10-years OS was recorded at 0.75 (95% CI = 0.69–0.82), 0.78 (95% CI = 0.73–0.82), and 0.77 (95% CI = 0.74–0.81), respectively (Figure 3). The C-index of this model was 0.71 (95% CI = 0.68–0.74). After 1000 bootstrap resamples, the adjusted C-index was 0.70. The calibration curves after 1000 bootstraps showed a good agreement between the actual and predicted values (Figure 4). The median total scores of all patients were recorded at 130.3. We divided patients into high-risk and low-risk groups according to the median points, and used the Kaplan–Meier curve with a log-rank test to examine the group differences. The median survival time in the high-risk groups was 90. The Kaplan–Meier curve (Figure 5) illustrated that this model has good performance in identifying the population with different risk levels (*p*<.0001). DCA curves showed that the model had a net benefit across about 60%, 80%, and 90% of the range of risk threshold in 3-years, 5-years, and 10-years, respectively (Figure 6).

#### 3.3.2. External validation

Good calibration was observed for the survival probability in the validation cohort (Figure 4). The AUC of 3 years, 5 years, and 10 years OS was 0.76 (95% CI = 0.68–0.83), 0.79(95% CI = 0.73–0.85), and 0.80 (95% CI = 0.76–0.85), respectively (Figure 3). The C-index of the nomogram for the prediction of OS was 0.72 (95% CI, 0.69–0.76).

## 4. Discussion

Given the high heterogeneity between CKD populations, identifying high-risk individuals for interventions not only improves patients adverse outcomes but also helps reduce the medical burden. Previous research [17] has primarily focused on predicting OS in dialysis patients. For non-dialysis CKD patients, an accuracy prediction tool is equally important. Therefore, we conducted a prediction model to calculate the OS of patients with moderate to severe CKD, and then visualized it through the use of a nomogram. The results revealed that this nomogram demonstrates a good performance in its ability to distinguish between high and low-risk stage 3–5 CKD patients. The prognosis of patients with moderate to severe CKD was also accurately predicted, which meant that the established model can be accurately used in clinical practice. The data of follow-up time and survival situations were retrieved from the NHANES database (2001–2004 survey). We integrated the follow-up data and indicated that the rate of death for patients with stage 3–5 CKD in 3 years, 5 years, and 10 years was 13.18%, 22.02%, and 48.9%, respectively. These results were similar to a nationwide study conducted in SWEDEN [18], which investigated the influence of sex-specific differences for CKD progression and mortality. The cause of the most common death was attributed to cardiovascular complications, and this factor has been solidified through general consensus [2]. The European Atherosclerosis Society (EAS) guidelines for dyslipidemia classified patients with CKD stage 3 as a high-risk population for progressing in chronic heart diseases, whereby the patients with stage 4 are defined as a very high-risk population [19]. In combination with our findings, the importance regarding the prevention of cardiovascular complications for patients with stage 3–5 CKD is re-emphasized. Based on the literature reviewed, as well as clinical experience, a total of 17 possible prognostic indicators of CKD were included. UACR, CKD stage, Cys C, age, potassium, and homocysteine were utilized to build the prediction model after screening for predictors by the Lasso regression. UACR is recognized as an independent risk factor for the prognosis of CKD. Previously, a prospective community-based cohort study [20] included 4883 subjects aged ≥20 years showed that high levels of UACR were associated with a high risk of all-causes of death. Another cohort study [21], which is based on the NHANES database, enrolled 31,413 patients with normal range albuminuria and found that elevation of UACR within the normal range also increases the risk of all-causes of mortality. The nomogram in our work demonstrated that the presence of albuminuria in CKD patients can be associated with poor survival outcomes, even in patients with microalbuminuria. Serum Cys C is a sensitive indicator for evaluating renal function in patients with CKD, especially in stage 3–4 patients. A single-central cohort study [22] found that Cys C may be a useful predictor for predicting adverse kidney outcomes in patients with stage 3–4 CKD. Similarly, the present study attested to the fact that Cys C was a key factor in predicting all-causes of mortality in patients with advanced CKD. These results underscore the importance of clinical screening of Cys C to improve the understanding of prognosis of patients. The aging of the population is an important factor involved in the increased incidence of CKD [23]. There is broad agreement that eGFR declined as age increased, whereby the risk of death also significantly increased. Our study not only confirmed this view, but also showed that age was the leading cause of all causes-mortality, since age accounts for a majority of the nomogram scores. Therefore, the progression of adverse outcomes should be further emphasized in elderly patients with CKD.

Electrolyte disorders are common in patients with CKD, especially those diagnosed with stage 3–5. Furthermore, electrolyte detection is extremely necessary for patients with CKD as a quantitative clinical marker. Ultimately, fluctuations in serum potassium are of particular concern to clinicians. The SCREAM trial [24], which enrolled 78,997 patients with stage 3–5 CKD, indicated that increased serum potassium levels were associated with poor survival outcomes. Interestingly, this study also found that patients with advanced CKD appeared to have better potassium tolerance. Given the limitations of the study design, our study failed to specifically analyze the relationship between serum potassium level and death prognosis of patients with different stages of CKD. Nevertheless, our findings demonstrated that hyperkalemia posed serious risks of death. This suggested the importance of monitoring and controlling serum potassium in patients with stage 3–5 CKD. In addition to the above parameters, our study found that patients with serum homocysteine levels >12 umol/L experience an increased risk of death. This particular result denotes that taking 12 umol/L as the threshold of prognosis displays certain clinical significance. Serum homocysteine is generally elevated in CKD patients, especially those with ESRD. Higher homocysteine levels may aggravate the progression of cardiovascular and cerebrovascular adverse outcomes. An observational cohort study [25], based on the data from the NHANES database, attested to the fact that homocysteine levels could interactively affect the risk of all-causes mortality among the middle-aged and older populations. Given the poor prognosis caused by homocysteine, some studies have proposed that folic acid can be used to reduce the level of homocysteine and improve the prognosis, although this idea remains controversial.

It is noteworthy that CKD stage and diabetes were both identified as important factors for the prognosis of CKD [26–28]. Nonetheless, in our study, the Lasso regression did not screen them as the key variables. After including them as the predictors in multivariate Cox regression, we discovered that there was no significant difference in mortality between patients in stages 3a, 3b, and 4. Patients with stage 5 CKD, however, displayed a considerably higher risk of death. In light of this, we added CKD stage to the nomogram as a prognostic factor. In contrast, diabetes was not linked to all causes of death, even in multivariate Cox regression. This could be attributed to the fact that some of the main determinants of survival (such as Cys C and potassium) are relatively low in our diabetes patient sample, so the impact of diabetes on all causes of death was offset.

This nomogram is based on the laboratory tests and census data obtained from the NHANES database. We included the prognosis factors which were commonly used in clinical practice. Therefore, it facilitates uncomplicated, efficient assessment for nephrologists. The external validation also proved that this nomogram could be applied to a variety of patients. Nonetheless, the limitations of this study also need to be addressed. First, although we did our best to include indicators that might be associated with CKD prognosis, some predictors such as neutrophil gelatinase-associated lipocalin or kidney injury molecular-1 were not included due to the limited data of the NHANES database. Second, our nomogram's prognosis was primarily based on laboratory indicators. Some prediction elements, such as nutrition, environment, and genetics, are also highly essential. Third, we performed external validation using patients from the NHANES database at different time points. However, multicenter clinical validation is also required to further evaluate the external ability of the nomogram.

## 5. Conclusions

This study demonstrated that age, UACR, Cys C, potassium, CKD stage, and homocysteine could be significant predictors of all causes of death in patients with stage 3–5 CKD. Additionally, a simple and practical nomogram demonstrating good discrimination and calibration has been effectively established to accurately predict the OS of patients with stage 3–5 CKD.

## Author contributions

NL and LZ contributed to the concept and design of this study. NL, GWZ, and YWZ were responsible for statistical analysis and writing of the report, WMH assisted in statistical analysis, ECZ and WS reviewed the article and provided critical feedback to improve and structure the report. All authors read and approved the final manuscript.

## Data Availability

The datasets used and analyzed in this study are available from the first author and corresponding author on reasonable request.

## Abbreviations

CKD: Chronic kidney disease
UACR: Urinary albumin-to-creatinine ratio
Cys C: Cystatin C
OS: Overall survival
eGFR: Estimated glomerular filtration rate
HbA1c: Glycosylated hemoglobin
BUN: Blood urea nitrogen
UA: Serum uric acid
CRP: C-reactive protein
DCA: Decision curve analysis
C index: Concordance index
ROC: Receiver operating characteristic
AUC: Area under the receiver operating characteristic curve
LASSO: Least absolute shrinkage and selection operator
NHANES: National Health and Nutrition Examination Survey
NCHS: National Center for Health Statistics
IRB: Institutional Review Board
